# Generalized Poincaré Plots-A New Method for Evaluation of Regimes in Cardiac Neural Control in Atrial Fibrillation and Healthy Subjects

**DOI:** 10.3389/fnins.2016.00038

**Published:** 2016-02-16

**Authors:** Mirjana M. Platiša, Tijana Bojić, Siniša U. Pavlović, Nikola N. Radovanović, Aleksandar Kalauzi

**Affiliations:** ^1^Faculty of Medicine, Institute of Biophysics, University of BelgradeBelgrade, Serbia; ^2^Laboratory of Radiobiology and Molecular Genetics, Institute of Nuclear Sciences “Vinča,” University of BelgradeBelgrade, Serbia; ^3^Faculty of Medicine, Pacemaker Center, Clinical Center of Serbia, University of BelgradeBelgrade, Serbia; ^4^Department for Life Sciences, Institute for Multidisciplinary Research, University of BelgradeBelgrade, Serbia

**Keywords:** Poincaré plot, autonomic nervous system, heart rate, atrial fibrillation, indexes of asymmetry, neural control regimes

## Abstract

Classical Poincaré plot is a standard way to measure nonlinear regulation of cardiovascular control. In our work we propose a generalized form of Poincaré plot where we track correlation between the duration of *j* preceding and *k* next *RR* intervals. The investigation was done in healthy subjects and patients with atrial fibrillation, by varying *j*,*k* ≤ 100. In cases where *j* = *k*, in healthy subjects the typical pattern was observed by “paths” that were substituting scatterplots and that were initiated and ended by loops of Poincaré plot points. This was not the case for atrial fibrillation patients where Poincaré plot had a simple scattered form. More, a typical matrix of Pearson's correlation coefficients, *r*(*j*,*k*), showed different positions of local maxima, depending on the subject's health condition. In both groups, local maxima were grouped into four clusters which probably determined specific regulatory mechanisms according to correlations between the duration of symmetric and asymmetric observed *RR* intervals. We quantified matrices' degrees of asymmetry and found that they were significantly different: distributed around zero in healthy, while being negative in atrial fibrillation. Also, Pearson's coefficients were higher in healthy than in atrial fibrillation or in signals with reshuffled intervals. Our hypothesis is that by this novel method we can observe heart rate regimes typical for baseline conditions and “defense reaction” in healthy subjects. These data indicate that neural control mechanisms of heart rate are operating in healthy subjects in contrast with atrial fibrillation, identifying it as the state of risk for stress-dependent pathologies. Regulatory regimes of heart rate can be further quantified and explored by the proposed novel method.

## Introduction

Standardized Poincaré plot of the first order is a graphical representation of temporal correlations within time series of inter-beat intervals in which an *RR* interval is plotted against its first predecessor. Generally it is the measure of nonlinearity in heart rate (HR) neural regulatory systems. Classically, a standardized Poincaré plot can quantitatively be evaluated by two measures of variability, i.e., two measures of standard deviation: SD1, the measure of variability across the line of identity, measuring how big the difference in duration of two successive *RR* intervals can be; SD2, on the other hand, is the measure of variability along the identity line, measuring how dispersed successive *RR* intervals of equal or similar durations can be (Guzik et al., 2006; Porta et al., 2008). Their major contribution is in the field of recognition of different types of cardiac arrhythmias (Zhang et al., 2015), dilated cardiomyopathy (Voss et al., 2012), and in the research of physiology of aging and gender (Voss et al., 2015).

Nonlinearity is a well-known characteristic of HR regulatory systems. In physiological circumstances, different cardiovascular (Eckberg, 1980; Ottesen and Olufsen, 2011) and extra cardiovascular systems (Wu et al., 2005; Kapidžić et al., 2014) influence its dynamics. In physiological situations (Delaney and Brodie, 2000), and pathophysiological situations, like heart rate arrhythmias, nonlinearity of HR changes in a specific manner, making it possible to distinguish different types of arrhythmias. Poincaré plot is a typical example of presenting how these nonlinearities are manifested (Zhang et al., 2015).

Atrial fibrillation (AF) is a sympathovagally triggered disease with dominant vagal role in the initiation of a paroxysmal episode (Chou and Chen, 2009). It is one of the pathophysiological models where altered neural control can be observed and evaluated. Once initiated, it is characterized by multifocal atrial electrical activity that irregularly passes through atrioventricular conductive pathway and depolarizes the ventricules. Atrial fibrillation is a typical neurocardiovascular disease with specific heart rate rhythm pattern but the specificities of autonomic remodeling that takes place in this pathology are still unknown. It is known that increased sympathetic innervation is present in patients with persistent AF, testifying that autonomic remodeling is present. In order to evaluate common functional modulation of both sympathetic and parasympathetic branches of cardiac autonomic nervous system, in the sense of a “black box” system, we applied a novel generalized modality of Poincaré plot in healthy and AF subjects. This is the first time that the generalized Poincaré plot (gPp) is proposed and in order to test its potential, we applied it on healthy subjects and AF patients.

## Methods

### Subjects

Ethic Committee of the Faculty of Medicine, University of Belgrade approved this study. All subjects gave written informed consent in accordance with the Declaration of Helsinki. Ambulatory patients with permanent atrial fibrillation (mean age 73; range 51–89) were included. Control subjects were gender matched, 10 men and 3 women. Control group were healthy middle aged subjects (mean age 41; range 35–45 years).

### Data acquisition

Measurements were done in the morning between 9.00 and 12.00 a.m. Subjects were supine with spontaneous breathing during 20 min of ECG measurements (without moving and verbal communications). The ECG was acquired with sampling frequency of 1 kHz by Biopac MP100 system with AcqKnowledge 3.9.1. software (BIOPAC System, Inc., Santa Barbara, CA, USA). ECG data were collected using 100C electrocardiogram amplifier module, leads and on subjects applied AgCl electrodes—Lead I. *RR*(*t*) inter-beat intervals was extracted from ECG using OriginPro 8.6 (OriginLab Corporation, USA), visually checked and manually corrected if necessary.

### Generalized poincaré plots

Further analysis was done with our original programs developed within MATLAB 6.5 (MathWorks Inc., Natick, MA 01760-2098 United States). In the following text, *RRn*−*j* refers to summed duration of previous successive *j RR* intervals, while *RRn*+*k* denotes the same quantity for the next successive *k* intervals. Both quantities were calculated by simply adding the durations of the corresponding intervals around a chosen *R* wave which was moving along the ECG signal. However, a natural limitation imposed on the number of points in these generalized Poincaré plots had to be observed: for an ECG signal with a total of *N RR* intervals, only *N – j – k* points could be drawn. In order to differentiate results obtained with specific values of *j* and *k*, for a pair of number of intervals, *(j,k)*, we propose the term “order of the gPp.” Increased complexity of gPp scatter grams, compared to classical Poincaré plots, allows one to study their different properties. In this work we concentrated mostly on their visual characterization and on the resulting Pearson's coefficients of linear correlation *r(RRn*−*j,RRn*+*k)*.

While in case of classical Poincaré plots only one value for each ECG recording is obtained, here we were dealing with matrices *r(RRn*−*j,RRn*+*k)* which we briefly denoted as *r(j,k)*. Out of many possible characteristics of these matrices, we were interested in the asymmetry of their element values, since we noticed that this property was very sensitive to the state of patient's health. In order to quantify it, we introduce a normalized asymmetry index (*NAI*), which for a *m* × *n* type matrix is defined as

$$NAI=\frac{1}{m\times n}\frac{1}{\bar{r}}\sum_{j\;=\;1}^{m}\sum_{k=j\;+\;1}^{n}\left(r\left(k,j\right)-r\left(j,k\right)\right)$$

where *r(j,k)* represents matrix element, while

$$\bar{r}=\frac{1}{m\times n}\sum_{j\;=\;1}^{m}\sum_{k\;=\;1}^{n}\left|r\left(j,k\right)\right|.$$

By introducing this particular type of normalization, we were able to compare asymmetry indexes calculated from matrices of different range of their element values, as well as their different sizes.

Another property of these Pearson's matrices which drew our attention was the appearance and positions of local maxima, since each local maximum of correlation could potentially signify a temporal range in which a neurocardial regulatory mechanism is operating. However, one should be very careful to verify that a physiological mechanism is lying beneath the appearance of a particular maximum, rather than any of numerous artifactual causes. It is not easy to separate these two causes, both for asymmetry and local maxima, but one of the approaches described in the literature is the method of random reshuffling of the detected *RR* intervals, which we adopted in this work (Guzik et al., 2006; Burykin et al., 2014). More, for each individual, *r(j,k)* matrices obtained for 10 repeated reshufflings were averaged and the corresponding *NAIsh* indexes calculated. Finally, the corrected version of an asymmetry index, *NAIC*, was obtained as their difference: *NAIC* = *NAI*−*NAIsh*.

### Statistics

Mann Whitney U test was used to compare indexes of asymmetry, *NAIsh* and *NAIC*, between healthy subjects and AF patients as well as their values in every group. To identify groups with different values of the Pearson's correlation coefficients maxima, a k-means clustering analysis was performed. As each local maximum was characterized by three coordinates: its position on the (*j*,*k*) plane and its value *r*(*j*,*k*), three dimensional clustering was performed according to these variables. The number of clusters was determined by two-step clustering procedure. Pearson's coefficients, as the third coordinate of cluster centroids, were compared between AF patients and healthy subjects, also by using the Mann Whitney U test. The data are given as mean values ± standard errors. A value of *p* < 0.05 was considered significant. Statistical analyses were performed using the software package SPSS Statistics (version 17.0, SPSS Inc., USA).

## Results

### Characterization of generalized poincaré plots

**Figure 1:**
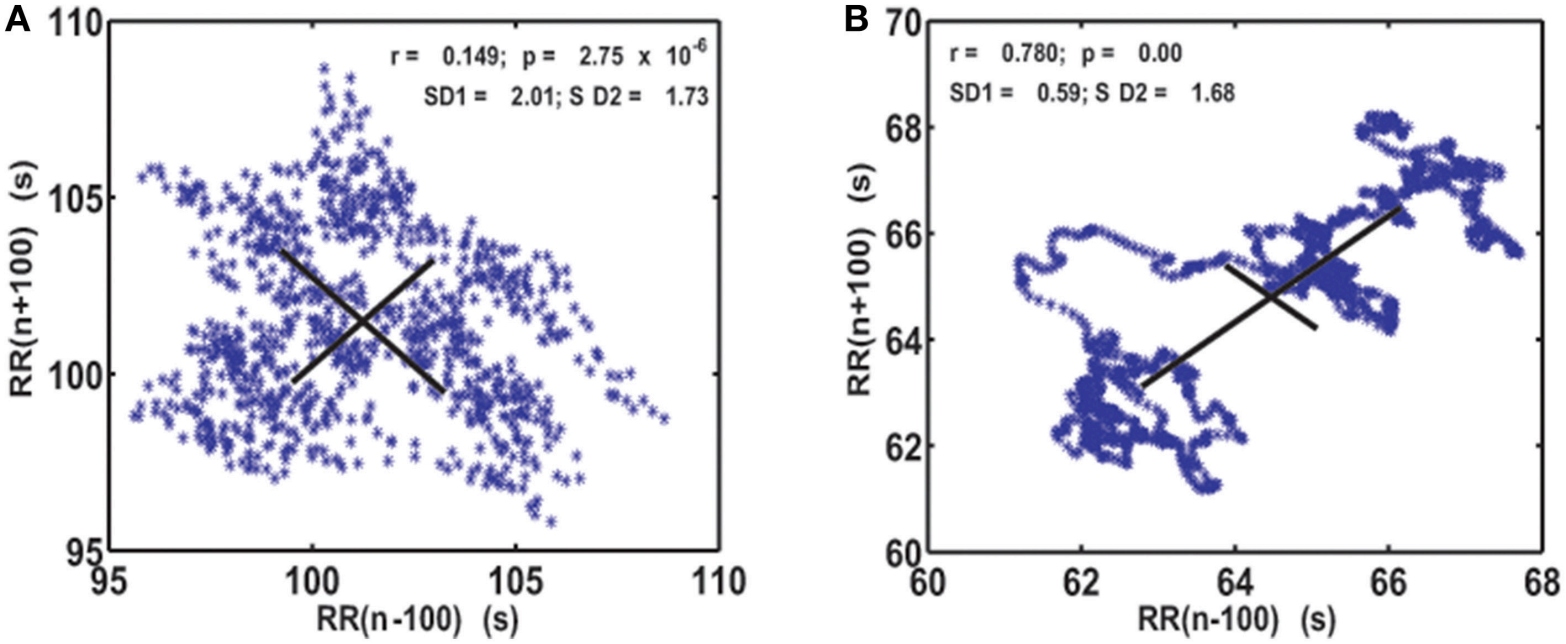
**Visualization of the system dynamics with generalized Poincaré plot for *j* = *k* = 100 *RR* intervals, in patient with atrial fibrillation (A) and healthy subject (B)**. *SD1* and *SD2*, drawn with solid lines, are measures of dispersion of 100 successive summed *RR* intervals along and away from the identity line.

As the first step of our analysis we calculated generalized Poincaré plots where *j* = *k* and (*j,k*) = 1, …, 100. In healthy subjects, as the order of gPp increased, classical Poincaré plots slowly changed into a more organized pattern. They were characterized by trajectories that were substituting scatterplots and which were initiated and ended by “hanks” or clustered points. This was not the case for AF patients, where Poincaré plots maintained their scattered forms (Figure 1).

**Figure 2:**
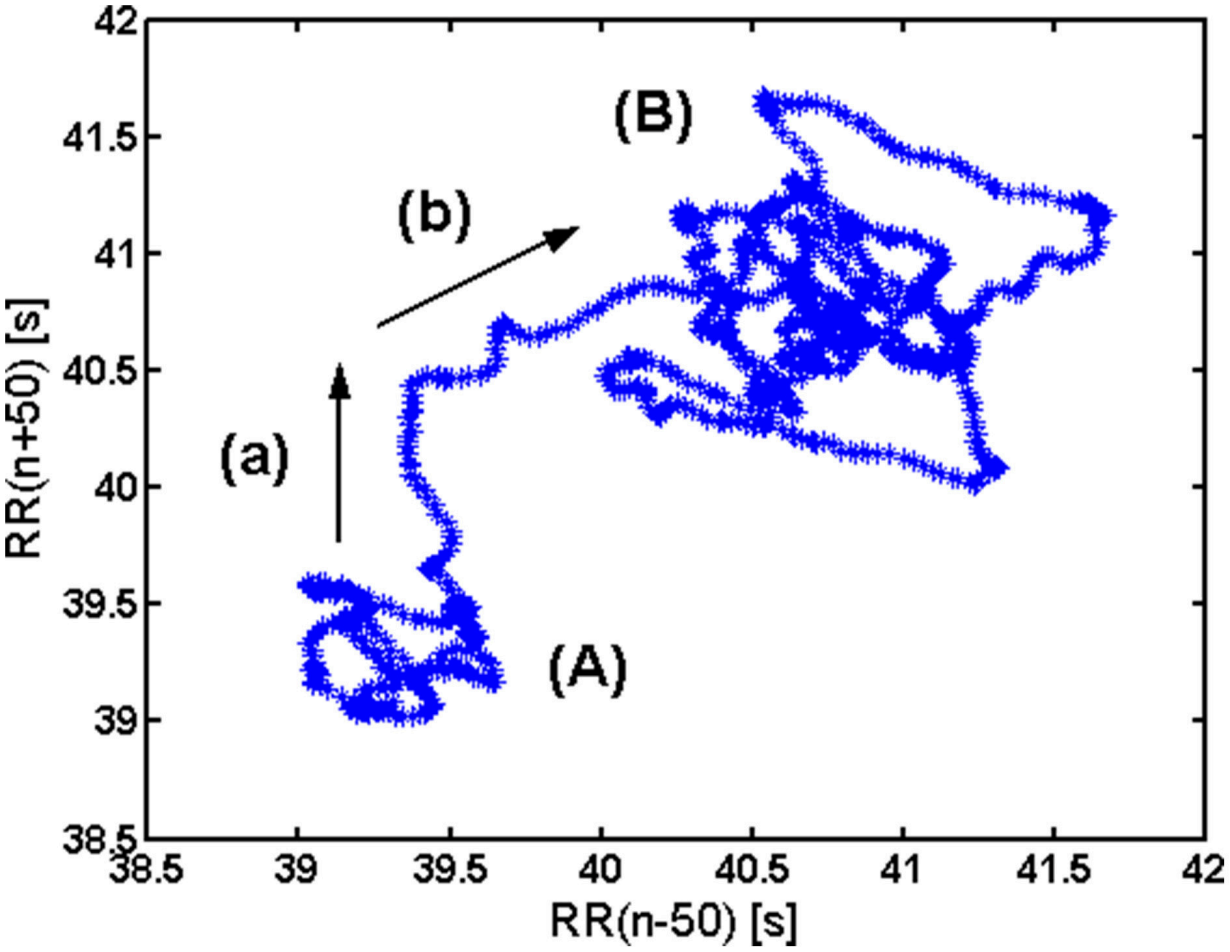
**Interpretation of elements of a generalized Poencaré plot in healthy. (A)** Tachycardic “hank”; **(B)** Bradycardic “hank”; (a) longer *RR* intervals entering the analysis; (b) shorter *RR* intervals exiting the analysis.

In healthy subjects, in basal conditions, two subregimes can be observed in a gPp (Figure 2). One zone, denoted with **(A)**, corresponds to a tachycardic regime, while the other one **(B)** corresponds to bradycardic regime. A two-phase transition from **(A)** to **(B)** is also visible: along (a) longer *RR* intervals enter the analysis, while along (b) shorter *RR* intervals exit the analysis window.

### Analysis of *r(j,k)* matrices

**Figure 3:**
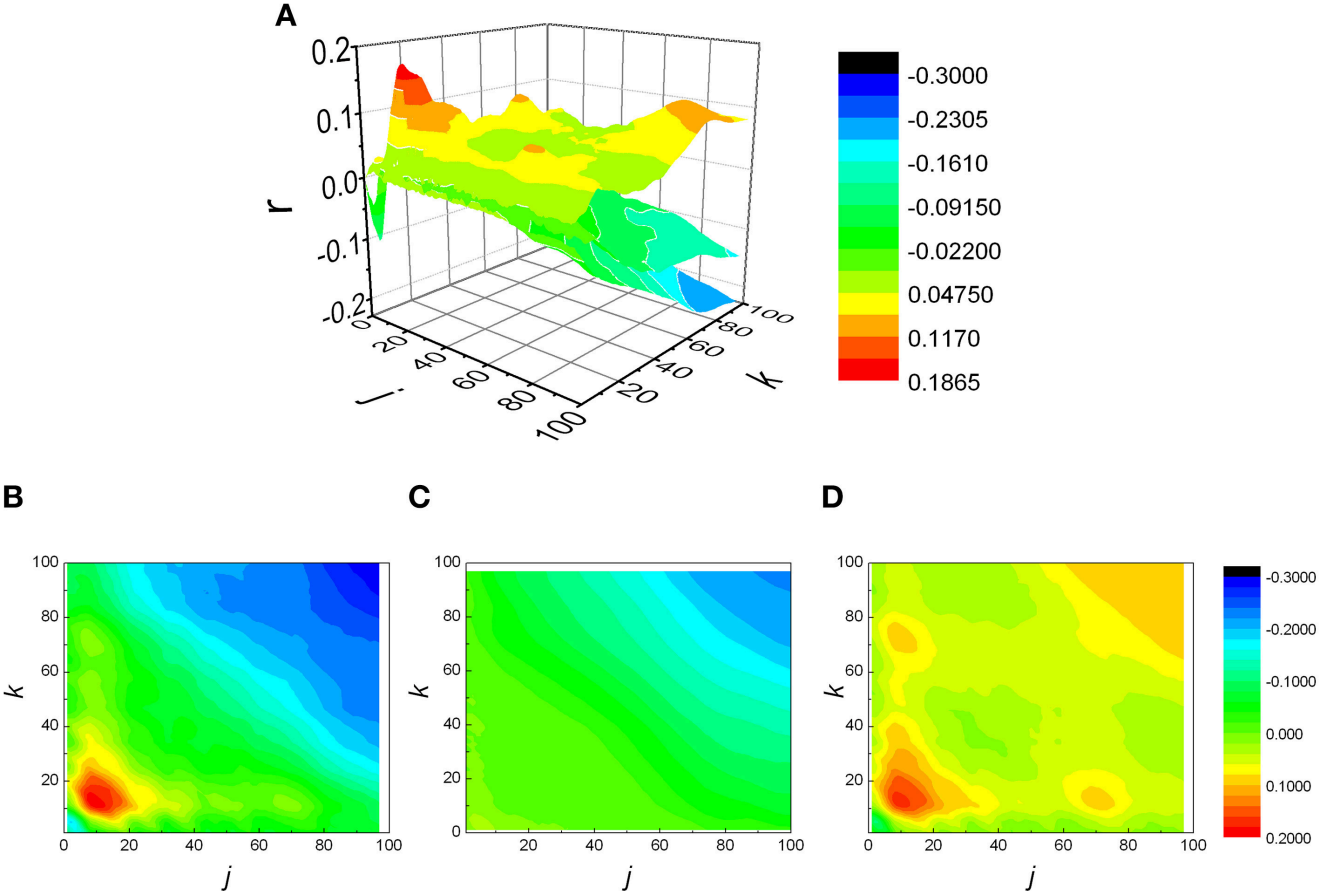
**(A)** Examples of three matrices of Pearson's correlation coefficients (*r*) between *k* following and *j* preceding *RR* intervals: physiological **(B)**, reshuffled **(C)**, and corrected **(D)** for generalized Poincaré plot of the 100th order in patient with atrial fibrillation.

**Figure 4:**
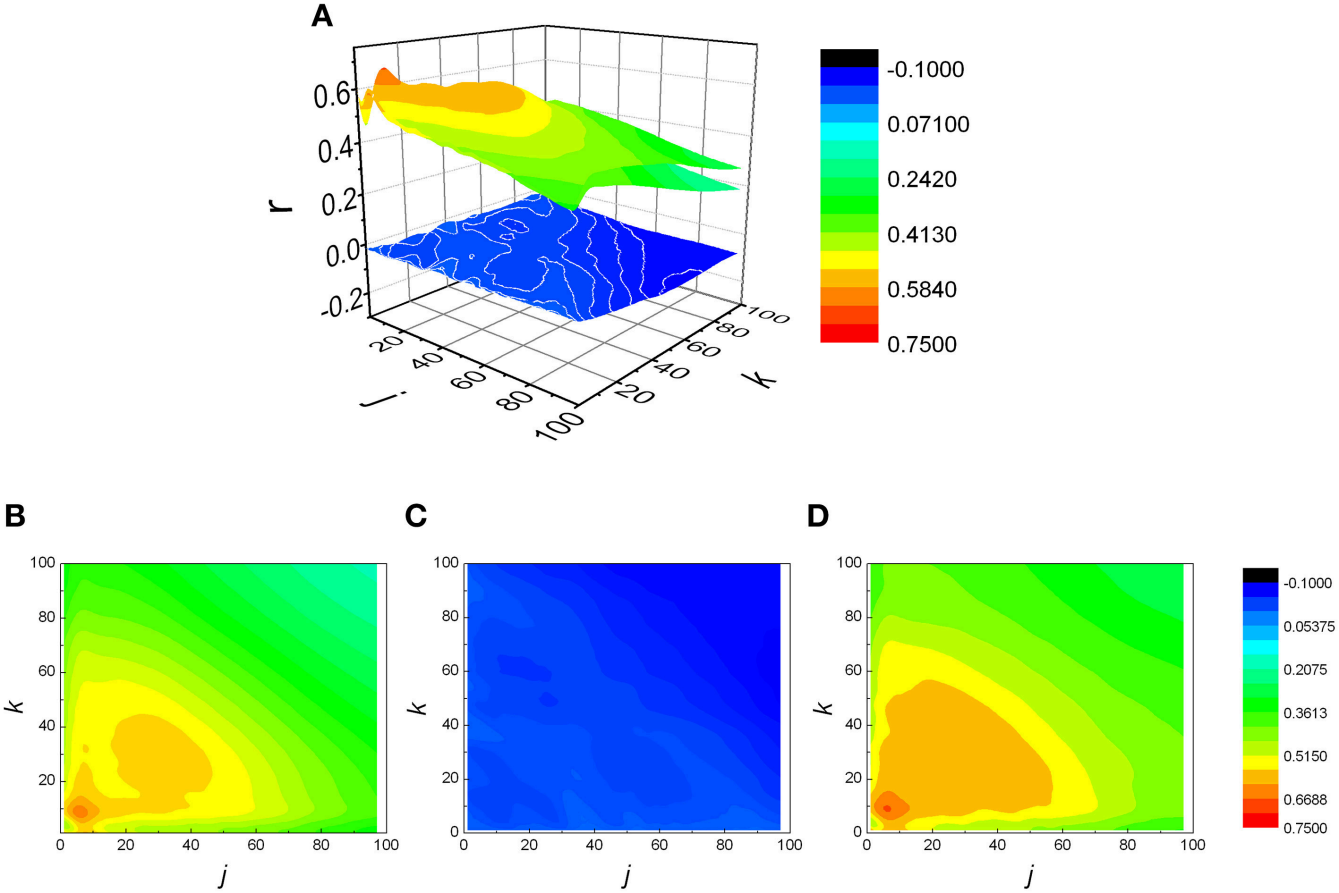
**(A)** Examples of three matrices of Pearson's correlation coefficients (*r*) between *k* following and *j* preceding *RR* intervals: physiological **(B)**, reshuffled **(C)**, and corrected **(D)** for generalized Poincaré plot of the 100th order in healthy subject. Physiological and corrected matrices appear as one surface because *r*_*sh*_*(j,k)* ≈ 0.

The analysis was expanded to *j* ≠ *k*, resulting in a matrix of Pearson coefficients *r(j,k)*. The reshuffled data were also analyzed and compared with measured data, generating *r*_*sh*_*(j,k)* matrices. Examples of two typical *r(j,k)* matrices, their reshuffled counterparts, *r*_*sh*_*(j,k)* and their differences *r(j,k)* − *r*_*sh*_*(j,k)*, are presented on Figures 3, 4.

**Figure 5:**
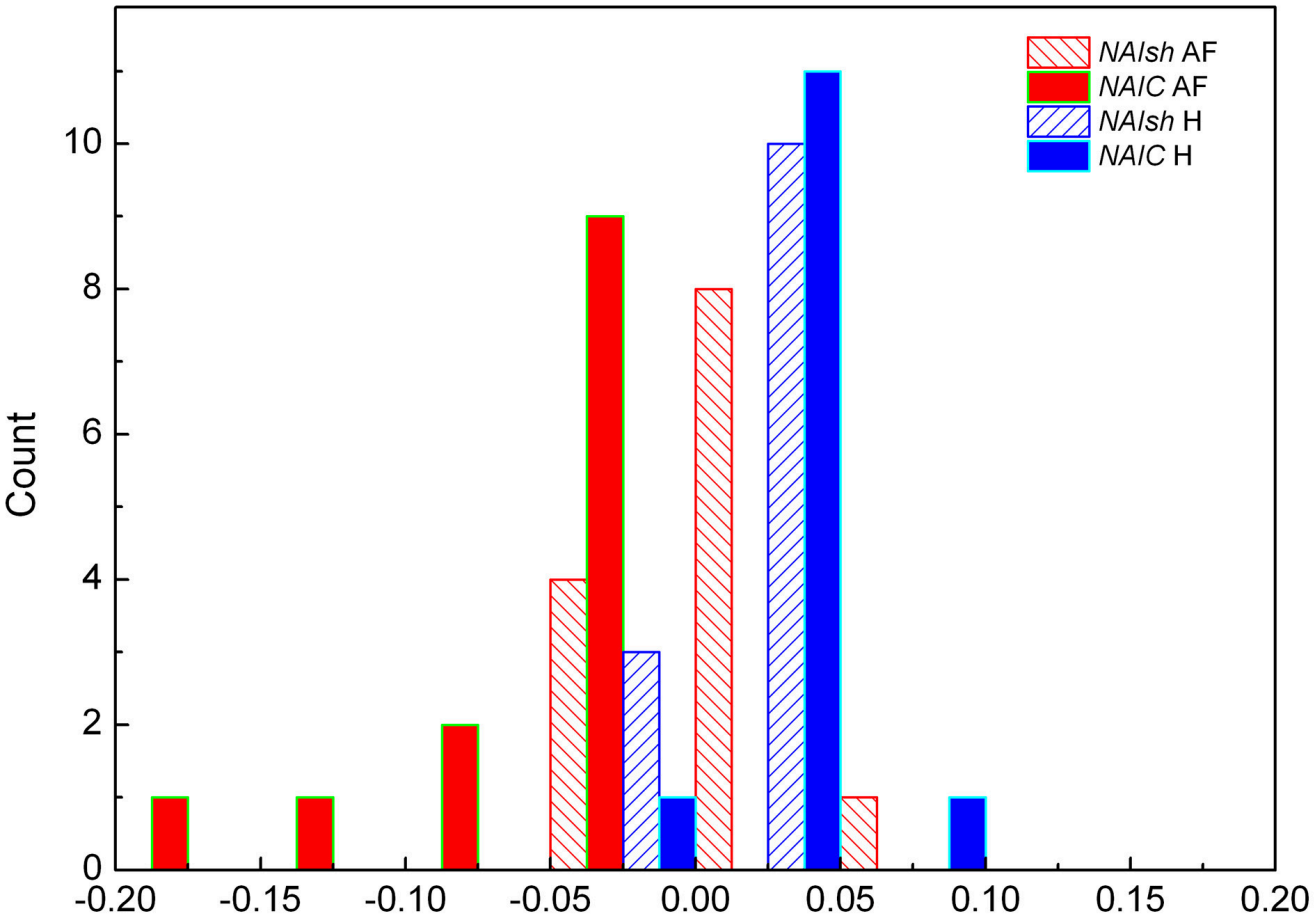
**Distributions of *NAIsh* (normalized index of asymmetry for *r*_*sh*_*(j,k)* matrices) and *NAIC* (corrected normalized index of asymmetry for *r(j,k)* matrices) in subjects with AF and healthy control H**.

For two groups of subjects we studied the distribution of index of asymmetry of reshuffled data *NAIsh* and corrected index of asymmetry *NAIC*, for *j,k* ≤ 100. In AF subjects the two distributions were sharply different (*p* = 0.007, Z = −2.691): for reshuffled data mean values of *NAIsh* were 0.0063 ± 0.0084, while all *NAIC* values were negative and asymmetrical in shape with mean values −0.047 ± 0.014 (Figure 5). As expected, the *NAIsh* was not different between groups (*p* = 0.801, Z = 0.778), but *NAIC* was (*p* < 0.001, Z = −4.283) (Figure 5). Their mean values for healthy were 0.0025 ± 0.0063 for *NAIsh*; 0.0138 ± 0.0056 for *NAIC*.

**Figure 6:**
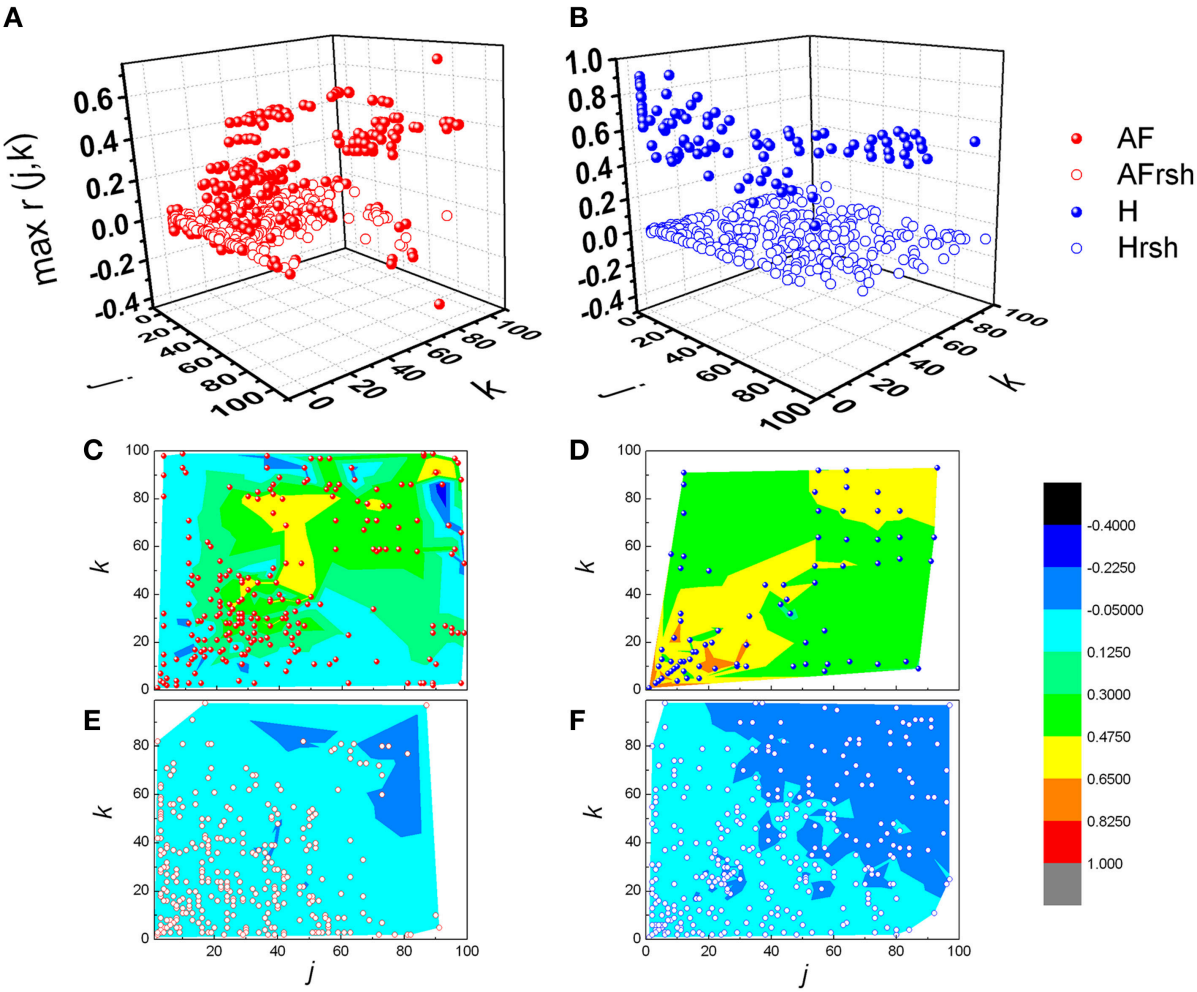
**Distribution of pooled local maxima of Pearson's correlation coefficient matrices – max *r*(*j*,*k*) in patients with atrial fibrillation (A) and healthy subjects (B) and their corresponding reshuffled data**. AF, AF patients; AFrsh, AF reshuffled; H, healthy; Hrsh, H reshuffled. Contour plots are given with corresponding maxima points for patients with AF **(C)** and their reshuffled data **(E)**, and healthy subjects **(D)** and their reshuffled data **(F)**. Contour plots are surface graphs of (*j*,*k*, max *r(j,k*)) data where ranges of max *r(j,k*) values are distinguished by different colors.

**Figure 7:**
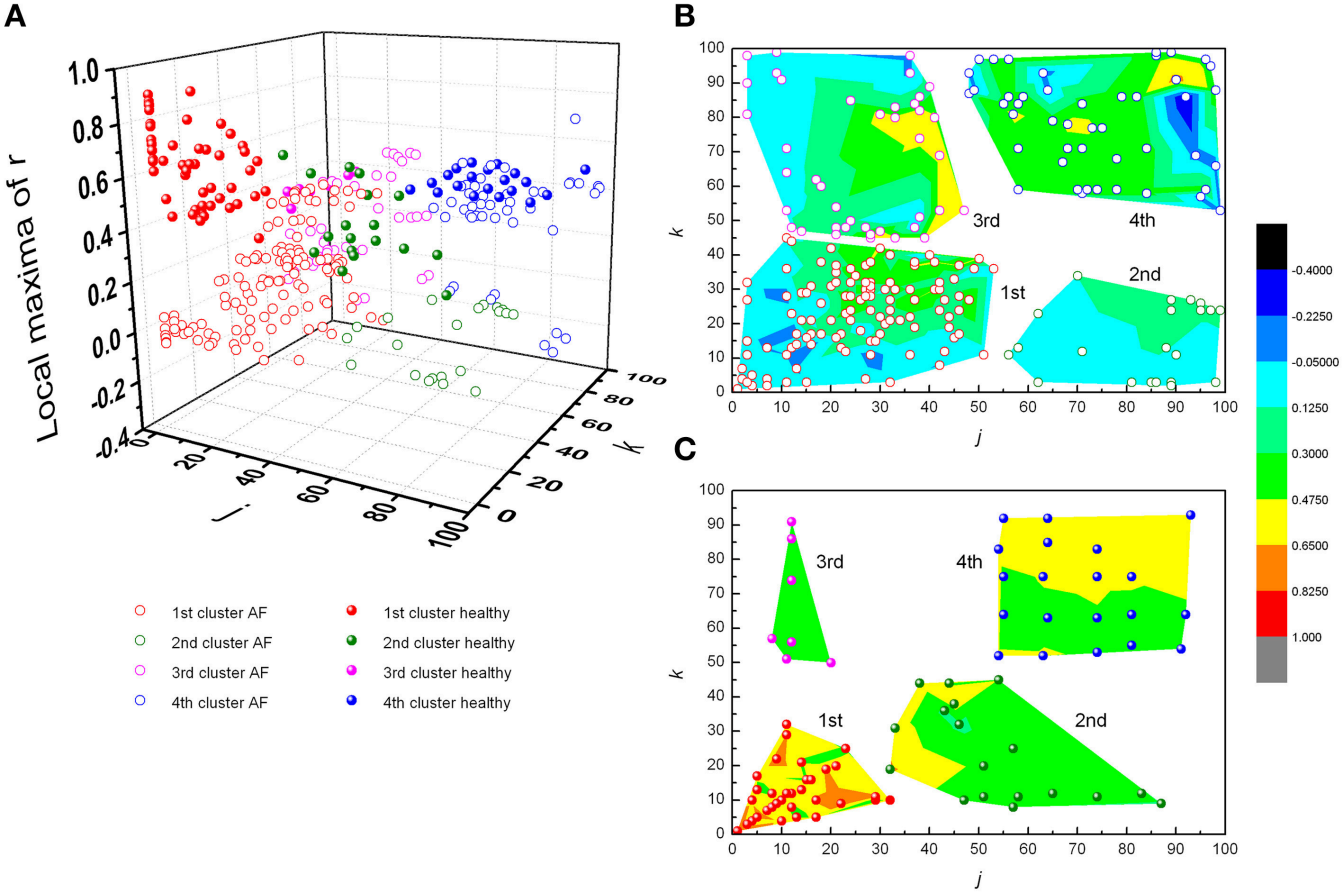
**Clusters of subjects' pooled local maxima of Pearson's correlation coefficients matrices – max *r*(*j*,*k*) in patients with atrial fibrillation and healthy subjects (A), and corresponding contour plots with local maxima points in patients with AF (B) and healthy subjects (C)**. Contour plots are surface graphs of (*j*,*k*, max *r(j,k*)) data where ranges of max *r(j,k*) values are distinguished by different colors.

From Figures 3, 4 it could be observed that local maxima of Pearson coefficients matrix were present, with different amplitudes and distributions both in AF patients and healthy subjects. Coordinates of all detected local maxima, from 13 AF patients, as well as from their 13 averaged reshuffled data, were measured and pooled. These data are presented as points in Figures 6A,C,E, while the same data for healthy subjects are drawn on Figures 6B,D,F. Coordinates of pooled local maxima for physiological (not reshuffled) data that were subjected to four cluster k-means algorithm are presented on Figures 7A–C.

The first cluster is located at position corresponding to a relatively low order of the Poincaré plot for both groups ((*j,k*) = 1, …, 50 in AF and (*j,k*) = 1, …, 30 in healthy subjects) but the values of local maxima of Pearson's coefficients in AF were significantly different from these values obtained in healthy subjects (Table 1). High values in healthy subjects and low values of *r* in AF indicate that correlation between summed durations of *RR* intervals in AF diminished in this short-range of observation. In healthy subjects, maxima with the highest values of Pearson's coefficients were located at *j,k* = 1 which corresponds to values for standardized Pp (not shown in Table 1). There is no statistical difference in maxima of Pearson coefficients between groups in the third cluster which is characterized by low correlation between large values of *k* and small values of *j*. Contrary, in the opposite second cluster, estimated for small values of *k* and larger values of *j*, significant correlation existed only in healthy subjects. Statistically significant difference between AF and healthy subjects was also found for maxima of Pearson's coefficients in the fourth cluster determined for *j*,*k* = 50, …, 100 (Table 1).

**Table 1:** **Mean values and standard errors of Pearson's correlation coefficients (*r*) between *k* and *j* intervals in clusters determined for patients with atrial fibrillation (AF) and healthy subjects**.

**Clusters**	**AF**		**Healthy**		***Z***	***p***
	**N**	***r***	**N**	***r***		
1st	121	0.161±0.016	50	0.627±0.021	−9.692	0.001
2nd	21	0.084±0.017	18	0.428±0.027	−5.324	0.001
3rd	41	0.227±0.033	7	0.388±0.014	−1.738	0.082
4th	46	0.296±0.034	20	0.4768±0.0013	−5.302	0.001

### Method validation using synthetic signals

In order to validate our method, a special MATLAB program was designed to generate a series of synthetic *RR* intervals in such a way that maximal correlation should be achieved for a given pair (*j,k*) of *j* preceding and *k* following intervals. When choosing initial parameters of this synthesis, we tried to imitate as much as possible the physiological values that were present in our subjects. By observing a typical histogram of measured *RR* intervals (not shown), we generated in our algorithm first *j*+*k RR* intervals as uncorrelated, by using a normal distribution, within the range 0.75–1.2 s (“randn” command in MATLAB). Next, an iterative scheme was programmed so that, with each step, duration of the next included interval was calculated so that the value of summed next *k* intervals tends to compensate the change in duration of the previous *j* intervals. However, if this compensation resulted in a value that violated the adopted range (0.75–1.2 s), the limitation posed by this range was applied as stronger, therefore introducing a desired degree of variability within the system.

**Figure 8:**
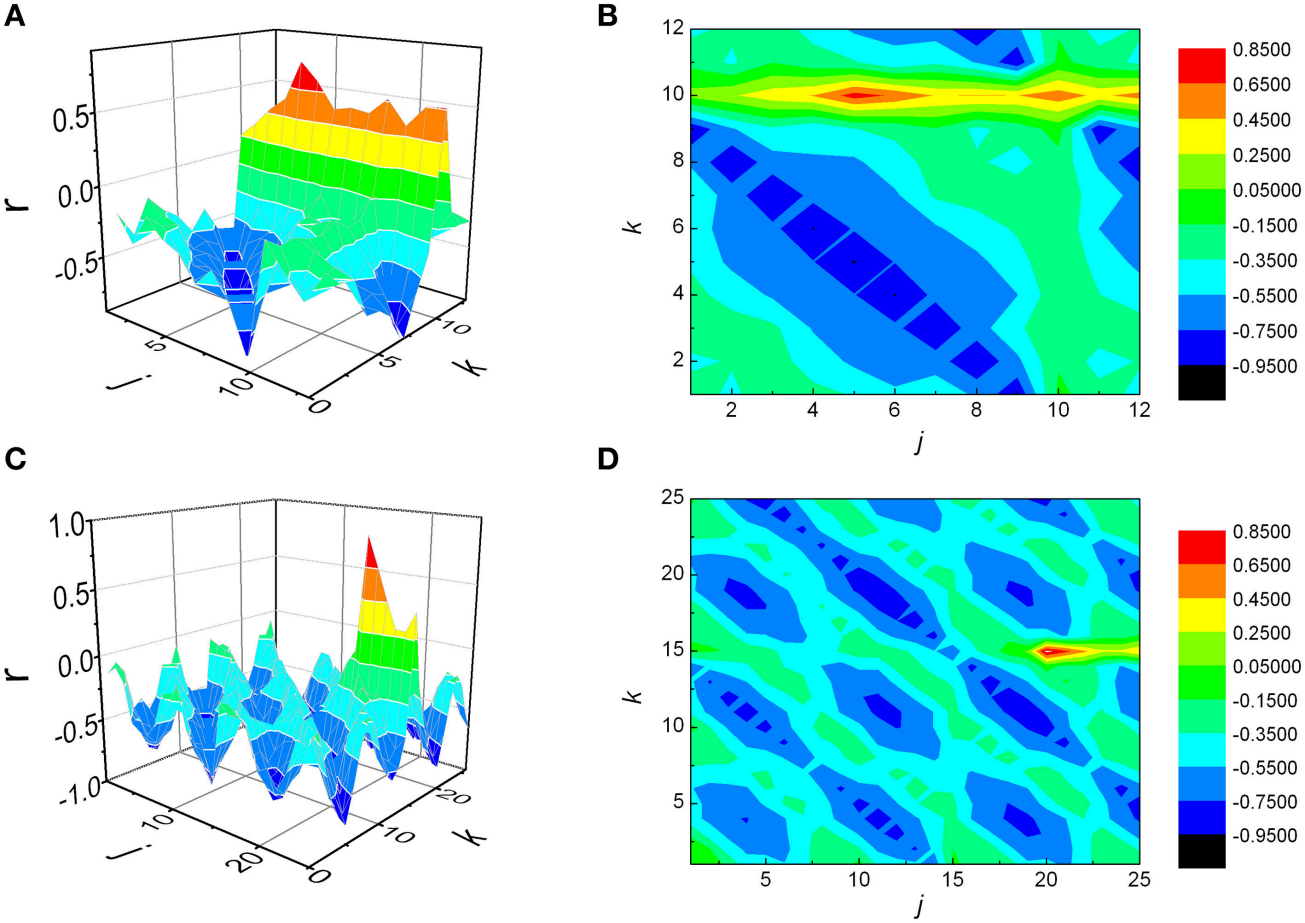
**Matrices of Pearson's correlation coefficients (*r*) between *j* preceding and *k* following *RR* intervals, obtained for two synthetic signals**. The first signal was generated in such a way that maximal correlation is to be achieved when summed *j* = 5, *k* = 10 intervals are being observed (case where *j* < *k*, **A** and **B**), while in case of the second one the values were *j* = 20, *k* = 15 (*j* > *k*, **C** and **D**). As presented, in both cases the analysis was able to detect correctly positions of *r*_*max*_.

We generated two series of data, by setting maximal correlation for *j* = 5, *k* = 10 (case where *j* < *k*) in the first synthetic signal, and *j* = 20, *k* = 15 (*j* > *k*) in the second example (Figure 8). Each signal was subjected to the same analytical procedure as our physiological data, and the results are presented on Figures 8A–D. As observed, in both cases maximal correlation was detected precisely at those values of *j* and *k* which were set prior to the analysis. Regarding the sign of *NAI*, as expected, it was negative (−0. 0723) in the first case, where maximal *r* was positioned above the identity diagonal, while a positive value (0.0380) was obtained in the second synthetic signal, where it was situated below this line.

## Discussion

It is well-known that blood pressure–heart rate baroreflex operates under different regimes, depending on the metabolic demand and different level of “central command” input (Sagawa, 1983; Rowell, 1993; McIlveen et al., 2001; Zoccoli et al., 2001; Bojić, 2003). This “baroreflex resetting” is visible both under the exercise (dominantly metabolic demand) and “defense reaction” (dominantly central command; Bauer et al., 1988; Jansen et al., 1995). These aforementioned drives change independently both the heart rate and arterial blood pressure set point (Sagawa, 1983; Bauer et al., 1988; McIlveen et al., 2001). By our novel analysis we are able to visualize different regimes (or “set points”) of heart rate control in the form of loops, which are delineating the areas of different regimes present in the conditions of basal metabolic demands. These regimes of HR regulation are connected with transitional “paths” connecting one HR regime with the other (Figure 2). It is plausible that in our baseline metabolic condition the HR regulatory system passes through different regimes due to different attentional and emotional states, psychological stress or even “defensive behavior” (in physiological terms—different levels and patterns of “central command”; Dampney et al., 2008; Peressutti et al., 2012). The parasympathetic control of heart rate, which is the dominant mechanism of heart rate control in basal conditions (Rowell, 1993) is shown to relate to emotional and attentional state of the subject (Porges, 1992; Suess et al., 1994; Thayer et al., 2009). The fact that “the regimes” were not registered in AF patients speaks for the presence of lower HR adaptiveness in these patients, especially in the circumstances of increased central command in different attentional and emotional states. The dominant adaptive mechanism to “defensive reaction” in AF patients is an increase of stroke volume by an increased cardiac contractility, while HR increase is less present (Goldstain, 2001). This maladaptive pattern creates the diathesis to hypertrophic cardiomiopathy and subsequent degenerative changes of myocardium (Goldstain, 2001). The maladaptive mechanism of AF in defensive reaction is dominantly an increase of cardiac contractility (an increase of stroke volume) and less an increase of heart rate. An increase of cardiac contractility by activation of beta adrenergic receptors increases intracellular concentration of calcium by an increase of voltage gated CaV1.2 channels. An increase of intracellular calcium through different intracellular mechanisms in time results with an increase in size of cardiomyocites and consequently, the thickening of the heart muscle. This condition, known as hypertrophic cardiomyopathy is characterized by serious structural and electrical abnormalities of the heart. It is known that hypertrophic cardiomyopathy coexists with AF (Kumar et al., 2015). The fact that distinct HR regimes lack in AF patients make this group of patients especially vulnerable for the more severe development of hypertrophic cardiomyopathy with respect to subjects with normal cardiac rhythm. This is especially valid if the AF patients are exposed to repeated and continuous stressful circumstances. Future studies need to address the question whether the absence of different heart rate regimes can be considered as the data having an AF diagnostic value.

Regarding the distribution of *NAIsh* and *NAIC* indexes presented on Figure 5, where AF patients exhibited negative and significantly lower *NAIC* values than in case of healthy or reshuffled data, it would be interesting to give at least a technical interpretation of the results. Let us observe the *r(j,k)* matrix and its identity diagonal (*j* = *k*). According to the *NAI* definition equation, *NAI* is negative if the sum of *r(j,k)* below the identity diagonal is less than the sum of *r(j,k)* above it, in the system of reference where *j* is on abscissa, *k* on the ordinate. In that case average correlation for *k* > *j* should be greater than the one for *k* < *j*. In other words, since *k* > *j* refers to shorter preceding and longer following summed *RR* intervals, negative *NAI* means that shorter preceding intervals are more correlated with longer following intervals than the other way round. An attempt to give this fact a physiological interpretation, on the other hand, is much more difficult. Probably some kind of memory mechanism is involved here, but details of this remain to be explored in our future studies. The fact is that both our groups were registered in basal conditions where, in healthy patients, 75% of heart rate control is under vagal influence. In AF patients we have strong sympathoexcitatory background even in basal conditions. We can only hypothesize that the asymmetry of *NAI* in AF can represent different dynamics of sympathetic withdrawal (negative *NAI*, shorter preceding and longer following summed *RR* intervals) versus the effect of sympathetic stimulation on heart rate control. It is necessary to emphasize that both branches of autonomic nervous system act in synergy and that vagal contribution to this phenomenon cannot be excluded. Further pharmacological studies are needed for pathophysiological evaluation of *NAI*.

According to distance between the observed number of *RR* intervals and local maxima of their Pearson's correlation coefficients, four different clusters were recognized. In the first cluster, in healthy subjects, two such maxima were found, one for *j, k* = 1 and one for *j, k*≈10. Since these findings were lacking in reshuffled data, we deduced that they were the result of physiological mechanism(s). These phenomena correspond to the dynamics of two dominant neural control mechanisms - parasympathetic and sympathetic. It is known that high values of Pearson's coefficients of correlation, in case of standardized Poincaré plots (*j, k* = 1), correspond to strong correlation between each two successive *RR* intervals, independently of their duration (equally for pairs of shorter or longer *RR* intervals). Parasympathetic control acts fast, is quite powerful (efficient) and can change heart rate within one heart beat (Eckberg, 1980; Levy and Martin, 1996). Due to different dynamics of neurotransmitter release, different intracellular effector molecular mechanisms and different mechanisms of neurotransmitter removal from neuromuscular synaptic cleft, sympathetic nervous system acts slowly with respect to the parasympathetic system (with delay of ~10 s, Rowell, 1993; Zoccoli et al., 2001; Bojić, 2003). On the basis of these data we hypothesize that the two positions of local maxima of Pearson's correlation coefficients might correspond to the zones of control of parasympathetic (*j, k* = 1) and sympathetic control (*j, k*≈10). In healthy subjects these two maxima are well defined, implying that both heart rate neural controls are operative, while in AF patients they are diminished. In the second cluster of Pearson's correlation coefficient maxima, defined for small values of *k* and larger values of *j*, significant correlation existed only in healthy subjects, while there was no significant difference between healthy and AF subjects in the third cluster characterized by weak correlations between small values of *j* and larger values of *k.* Again, absence of these correlations in reshuffled data and especially their asymmetry in AF patients indicates their physiological origin. According to the duration of observed *RR* intervals belonging to the third cluster, we can only conclude that here regulatory mechanisms with a slower response (in the range of a few minutes) are involved, which are again disturbed by AF. The fourth cluster of pooled subjects' local maxima of Pearson's coefficient was also influenced by AF and probably quantify very slow regulatory mechanisms with a response longer than 3 min, which include termoregulatory mechanisms, renin-angiotesin system, hormonal, metabolic, vagal influence, etc. (Task Force, 1996). In the absence of pharmacological identification of underlying functional mechanisms we can only speculate on the identity and characteristics of the AF Pearson's correlation coefficient maxima, but our approach clearly showed that the pattern of heart rate neural regulation in AF patients is highly distorted, shifted toward higher frequencies and acquired some characteristics of random pattern. However, it is important to emphasize that our results showed that high irregularity of heart rhythm in AF patients was present only in the range of short time scales (approximately shorter than 30 *RR*) when correlation between *RR* intervals didn't exist. But, in the range of larger scales (approximately larger than 30 *RR* intervals) correlations between *RR* intervals appeared. Correlations were asymmetrically distributed and in general smaller then in healthy subjects. The last feature indicates existence of specific residual determinism in AF patients' heart rhythm which probably originated from some kinds of slower regulatory mechanisms. Future pharmacological identification studies need to be done in order to clear these findings.

One more important limitation of our study is the fact that the control group and the experimental group were not age matched. The lack of age-matching could be serious bias in heart rate variability study. We could not age match the groups because it was impossible to create the control group in the range 51–89 years without some cardiovascular pathology. This finding is in accordance with World Heart Federation statement that cardiovascular disease becomes increasingly common with age (http://www.world-heart-federation.org). Inclusion of age matched control subjects with cardiovascular pathology would surely bias the results of our analysis, while, on the other side, we had an interest in heart rate pattern of AF, and there were no data in the literature that the AF pattern is age dependent. We created the control group that was in age category as close as possible to the age catergory of the experimental group. With this precaution on mind, we believe that we obtained the comparison of AF pattern with clear physiological pattern of control subjects and that obtained results can be interpreted as the result of physiological regulatory mechanisms in control group and their pathophysiological modulation due to AF in the patient group.

In our study, by a newly developed generalized Poincaré plot analysis, we gave some new insights into regulation mechanisms of heart dynamics. The proposed method revealed two phenomena: first, transient regimes of system dynamics in summed heart period time series of healthy subjects; second, asymmetry of correlations between *RR* intervals in patients with atrial fibrillation.

## Author contributions

MP study protocol, calculation, statistical analysis, and writing of the manuscript. TB study design, data interpretation, and writing of the manuscript. SP clinical examination and recruitment of patients. NR ECG recordings. AK developed the method, calculation, writing of the manuscript. All authors contributed to and have approved the final version.

### Conflict of interest statement

The authors declare that the research was conducted in the absence of any commercial or financial relationships that could be construed as a potential conflict of interest.
